# How Leaders’ Psychological Capital Influence Their Followers’ Psychological Capital: Social Exchange or Emotional Contagion

**DOI:** 10.3389/fpsyg.2019.01578

**Published:** 2019-07-12

**Authors:** Qishan Chen, Yurou Kong, Jun Niu, Wenyang Gao, Jieying Li, Miaosi Li

**Affiliations:** Guangdong Key Laboratory of Mental Health and Cognitive Science, Center for Studies of Psychological Application, School of Psychology, South China Normal University, Guangzhou, China

**Keywords:** psychological capital, social exchange, emotional contagion, work team, multilevel multiple mediation effect

## Abstract

Using a sample of 32 work teams (32 work team leaders and their 321 followers) in Chinese cultural context, this study investigated the relationships between leaders’ and their followers’ psychological capital and the multilevel multiple mediation effects of social exchange and emotional contagion. PsyCap questionnaire (PCQ), leader-member exchange scale, and the positive affect scale in the positive and negative affect scale (PANAS) were adopted to measure variables. A total of 430 questionnaires were distributed in 2014 and the response rates were 90.2%. Structural equation model and hierarchical linear model were applied to analyze the survey data. The results revealed that leaders’ psychological capital had a positive influence on their followers’ psychological capital. Leader-member exchange was the cross-level mediator between leaders’ psychological capital and their followers’. The cross-level mediating effect of leaders’ positive emotions perceived by followers was not significant. The results of this study extended the social exchange theory and enriched researches on leadership. The implication was discussed in details.

## Introduction

Psychological capital refers to a positive mental state in growth and development of individuals, which specifically manifests as efficacy, optimism, hope, and resilience (Luthans et al., 2007c). Psychological capital draws more and more attention as an important topic in organizational behavior and human resource management (Luthans et al., 2007c, 2015; Youssef-Morgan, 2014). It can be found that the existing research has witnessed remarkable progress on psychological capital, but there are still some deficiencies. Considering the research content, previous studies mainly considered followers’ psychological capital as a factor affecting output variables including work attitude and work behavior rather than a variable can spread from person to person. Only a few studies have investigated the interactive mechanism of psychological capital between team leaders and their followers. As far as research methods, existing studies mainly concerned about variables at the individual level but tended to overlook the possible relations between variables at different levels.

Two recent studies done by Walumbwa et al. (2010) and Ren et al. (2013) used the hierarchical linear model (HLM) to explore the cross-level interpersonal interaction of psychological capital. They found that leaders’ psychological capital could affect their followers’ psychological capital and therefore promote the followers’ organizational citizenship behavior as well as job performance. The findings suggested that leader-follower interaction played a positive role in improving individual performance. By using HLM, these studies expanded the psychological capital literature from the individual level to the group level, as well as enriched the study of interpersonal interaction within teams, which were of great significance.

However, Walumbwa et al. (2010) and Ren et al. (2013) left an important question unanswered: Why leaders’ psychological capital produces a positive influence on their followers’ psychological capital For this question, they gave some possible assumptions. Walumbwa et al. (2010) explained the mechanism that leaders’ psychological capital influences their followers’ psychological capital by social learning theory, emotional contagion theory, and social exchange theory. Apart from social learning theory, social exchange theory and emotional contagion theory are competitive and at the same time, they have totally different theoretical orientations. Because Walumbwa et al. (2010) and Ren et al. (2013) have not validated their ideas by collecting empirical data, the current research tries to test the two theoretical assumptions so as to study the problem that how leaders’ psychological capital interacts with their followers’ psychological capital.

The interpersonal interaction between leaders and their followers will be influenced by culture. Culture is a mental program shared by people who lived in the same environment. This means that culture is not an individual characteristic but something commonly programmed in people who have similar education and life experience. People in different groups, regions or countries have different cultures. These cultural differences can be characterized in different forms and dimensions, such as individualism vs. collectivism, power distances and so on (Hanson et al., 2012). Chinese society is a high power distance society, with a deeply rooted notion of hierarchy and obvious worship to power. Given the special of Chinese culture, we will investigate the relationship of psychological capital between leader and their followers’ psychological capital in Chinese cultural context.

The purpose of this study was to explore the following issue: whether the influence of leaders’ psychological capital on their followers is a result of social exchange or emotional contagion under the Chinese cultural background? And for this, we proposed a multilevel multiple mediation model: Leaders’ psychological capital influences their followers’ psychological capital via the multilevel mediation effect of social exchange which is represented by leader-member exchange and the multilevel mediation effect of emotional contagion which is represented by leader’s positive emotions perceived by followers. If both multilevel mediation effects were verified, it means that the interaction between leaders’ and their followers’ psychological capital is the result of both social exchange and emotional contagion. If only one multilevel mediation effect were verified, it would indicate that the influence of leaders’ psychological capital on their followers’ psychological capital is just a product of social exchange or emotional contagion.

### The Influence of Leaders’ Psychological Capital on Their Followers

The concept of psychological capital was put forward by Luthans et al. (2007c, 2015) based on distinguishing it from capitals such as economic capital, human capital and social capital. According to Luthans’s theory, psychological capital is a kind of positive mental state in growth and development of individuals, which specifically manifests as efficacy, optimism, hope, and resilience. Efficacy refers to the characteristic that one has confidence in his or her ability and can strive for success in facing challenging work. Optimism refers to the characteristic that one has rational knowledge about what he or she can and cannot do as well as has a positive attribution style for present and future success. Hope refers to the characteristic that one has perseverance for a clear orientation, and can adjust his or her approach when necessary. Resilience refers to the characteristic that when facing problems or adversities, one can recover quickly and transcend difficulties to succeed with persistence.

The concept of psychological capital has been investigated widely since it was published. A meta-analysis showed that psychological capital not only can improve employees’ job satisfaction, organizational commitment, and subjective well-being effectively but also increase employees’ organizational citizenship behavior, subjective and objective career success. Moreover, it can reduce employees’ turnover behavior and abnormal behavior such as work stress, job burnout (Avey et al., 2011b).

A work team is a group of individuals who have a shared responsibility for achieving a goal or completing a task. In order to achieve the goal or to complete the task, interpersonal communication is necessary between leaders and their followers, and among followers. In a work team, people’s psychological capital can influence each other easily, especially from leaders to their followers (Story et al., 2013). Luthans et al. (2010) conceptualize psychological capital as state-like constructs which are more stable than states like moods and emotions. The study of the test-retest reliabilities also shown that the psychological capital scale had relatively lower stability than trait-like constructs like personality and core self-evaluations (Luthans et al., 2007b). Previous studies also supported psychological capital is a state-like characteristic and can be developed and changed over time (Story et al., 2013; Chen et al., 2017).

Leaders play different roles and undertake diverse functions in varying developing stages of a team for achieving the team goal. For one thing, they need to improve their followers’ working skills and positive psychological qualities. For another thing, they need to create a positive atmosphere for promoting their followers’ efficacy and cooperation spirit (Kozlowski and Bell, 2003; Mathieu et al., 2014). Since to improve followers’ positive qualities (i.e., psychological capital) is one of the leaders’ missions, leaders usually would like to affect their followers by showing their own positive psychological capital. According to the social learning theory (Bandura, 1997; Walumbwa et al., 2010), leaders who show high levels of positive psychological capital often play a role model for their followers so that the followers can imitate leaders’ behaviors by observational learning. In this learning process, followers will observe and mimic leaders’ positive attitudes and behaviors, also showing such positive psychological capital. Based on social identity theory (Tajfel, 1982; Hogg, 2006), leaders’ psychological capital can also influence follower’s psychological capital via increasing followers’ organizational identification (Chen et al., 2017). When team leaders assign tasks and give instructions to their followers, leaders’ psychological characteristics will be conducive to form an advantageous team climate which can affect followers’ organizational identification and psychological capital gradually. Leaders with rich psychological capital are more active and energetic than leaders lacking psychological capital, so they have strong will to look for solutions to problems. Similarly, they have an optimistic outlook and are more likely to bounce back from adversity. Their positive work attitude usually leads to outstanding performance, thereby promoting their followers’ psychological capital (Yammarino et al., 2008; Avey et al., 2011a).

Therefore, this research assumes (H1): Leaders’ psychological capital plays a positive role in promoting their followers’ psychological capital.

### Cross-Level Mediation of Social Exchange

Social exchange theory maintains that the essence of social activities is the process of exchanging material and non-material resources. People are prone to seeking for balance and interest maximization when they establish and keep most of the social relations (Cook et al., 2013). Social exchange theory has become one of the most influential theoretical frameworks in organizational psychology. Two kinds of exchange often occur in organizational psychological research: the exchange between employees and organization (e.g., organizational support) and the exchange between leaders and followers (i.e., leader-member exchange).

Leader-member exchange is a kind of social relation build up by team leaders and their followers at work, which generally embodies in two forms. One is the legitimate economic exchange relationship within the scope of contract. The other is the social exchange relationship beyond the work contract which was set up based on mutual trust and loyalty (Dulebohn et al., 2012). According to the social exchange theory, the establishment of a relationship is a process that followers exchange individual hard work and loyalty for available benefits and social reward from their leader. But this kind of social exchange shall follow the norm of reciprocity.

Resources theory in social exchange made a classification of different social exchanges: (1) money, currency; (2) tangible products or materials; (3) service, labor behavior; (4) information including advice, opinion, instruction and so on; (5) status, an evaluation about one’s prestige, regard and reputation; (6) love, expression of regard, warmth, and comfort (Foa and Foa, 2012). People prefer exchanges that involve neighboring classes of resources. For instance, an organization can exchange money for staffs’ service, but not necessarily for their suggestions or affections to the organization. It is called the preference of neighboring.

According to the norm of reciprocity and the preference of neighboring in social exchange, if a leader, the team spokesperson, invests material assets such as reasonable salary, benefits, and performance bonus, followers will be more likely to provide task performance such as high-quality work or overtime. If the leader invests development resources such as growth opportunities, promotion, and participation in decision-making, followers will be more likely to provide contextual performance such as organizational citizenship behavior, organizational commitment in return. And if the leader invests positive psychological resources such as efficacy, hope, optimism, and resilience, followers will be more likely to exchange psychological capital with the leader as rewards (Peng et al., 2014).

To be specific, leaders who show high levels of psychological capital are more active in cultivating mutual trust and loyalty with their followers, so as to attain a higher quality of LMX. Within a high-quality relationship, leaders will provide various resources (i.e., levels of responsibility and decision making latitude) and concerns to support their followers developing professional abilities and psychological resources including psychological capital (Story et al., 2013).

Therefore, this research assumes (H2): Besides promoting followers’ psychological capital directly, leaders’ psychological capital can also affect followers’ psychological capital through the mediation of leader-member exchange indirectly.

### Cross-Level Mediation of Emotional Contagion

In daily life and work, people not only speculate about the emotional state of others by capture verbal and non-verbal information but also automatically mimic others emotional symbols, such as facial expressions, voices, postures, and movements. Ultimately, their emotions tend to be similar to or consistent with the emotions of people around them. This phenomenon is called emotional contagion (Hatfield et al., 1994; Tee, 2015).

Emotion is a state of arousal accompanied by facial and bodily changes, physical arousal, cognitive appraisal, subjective feelings, and behavior tendencies. As an internal experience, emotion can help individuals to build relations with others and to determine the suitability of their emotional response when experiencing emotional events. In this way, emotion serves to satisfy two basic human social needs: affiliation and social consensus (Niedenthal and Brauer, 2012). To realize the social function of emotion must depend on outward expression. Emotional expression is a dynamic process in which individuals express their inner experience through explicit behaviors. In social communication and interpersonal interaction, emotional interaction often occurs. As a result, individuals speculate about the emotional state of others by capturing the emotional symbols and mimic the emotional symbols automatically to make their emotions more consistent with other people.

Emotional contagion not only exists among individuals, but also exists in organizations and work teams (Barsade and Gibson, 2012; Collins et al., 2013). In a work team with frequent communication, facial expressions, movements, and voices of a member can be received by other members easily, so as to bring about emotional contagion. Emotional contagion in the organization can form a circular effect between deliverers and receivers: Emotions of the deliverer was passed to the receiver, and then be fed back to the deliverer again so that the emotion of the deliverer would be further strengthened. Through this circular process, emotions of a member will spread out in the group, and be reinforced gradually, thereby forming a homogenous emotional state and social cognitive state among members (Collins et al., 2013; Sy and Choi, 2013).

One thing worth noticing is that individual differences also exist in emotional contagion. Some people are more easily influenced by emotions others expressed, and some people are better at spreading their emotions. Emotional deliverer’s social status in the organization and relationship with others are two important factors relating to the effect of emotional contagion. One with high status and high interpersonal attraction is more likely to bring emotional contagion than a person with low status and low interpersonal attraction (Boiger and Mesquita, 2012). Leaders play an important role in the formation of group emotions. The role of leader in group emotion has been emphasized by some leadership theory, such as charisma leadership theory which believes that emotion is an important part of effective leadership. Teams with a leader who shows more positive emotions usually are equipped with more enthusiasm and striving spirit, less burnout and interpersonal conflict than teams without such a leader. The result is that the team can be more efficient with strong team cohesion (Sy et al., 2013). Leaders’ emotions cannot only influence followers in short term but also determine the whole atmosphere of the team in long term, so followers will be immersed in a positive or negative state of emotion for long. These research findings were verified both in natural situations and in highly controlled experimental conditions (Bono and Ilies, 2006; Ilies et al., 2013).

**FIGURE 1:**
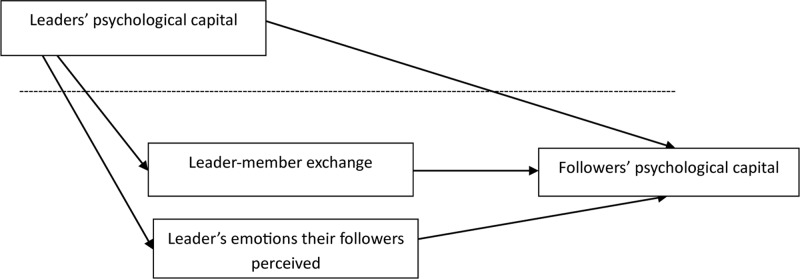
Cross-level mediation effect model of LMX and leader’s emotions their followers perceived.

Therefore, this research assumes (H3): Besides promoting followers’ psychological capital directly, leaders’ psychological capital can also affect followers’ psychological capital through the mediation of leaders’ emotions perceived by their followers indirectly. Hypothesis model is shown in Figure 1.

In order to cope with fierce competition, work teams become more and more important in organizations (Maynard et al., 2013). Team-based structures are common forms in modern enterprises (Rousseau and Aubé, 2018). Team leaders play a crucial role to shape team atmosphere, which further has a positive influence on their followers. Exploring the relationship of psychological capital between leaders and their followers helps us to understand how leadership transmits effectiveness to individuals. This study can enrich researches on leadership. On the other hand, to study the relation of psychological capital between leaders and followers from different theoretical perspectives, it is beneficial to enrich social exchange theory and emotional contagion theory and it is available to guide practical intervention about improving employees’ psychological capital in the future.

## Materials and Methods

### Participants

Participants were work teams from financial enterprises in different geographic areas in China selected by means of cluster sampling. They belong to the same industry, also with the same organizational form, work task, operating mode and performance assessment standard, which can minimize the influence of work characteristics, organizational culture and other factors on the research variables. With the assistance of senior managers, a total of 430 questionnaires were distributed to employees and their leaders directly, each work team with an independent code in order to match. A total of 388 questionnaires were collected, with response rates of 90.2%, of which 353 were valid questionnaires (from 32 work team leaders and their 321 followers), with 82.1% efficiency. The valid data were collected from 32 work teams (mean members = 10, range = 4–18). 94.7% of followers have kept leader-member relation with their direct leaders for at least 1 year, 2.85 years on average. The present study has been approved by the Research Ethics Committee of the respective university. We informed the participants about the general investigation information and got their signatures on the consent form before the measures. The data were collected and analyzed anonymously.

Among all the participants, 56.4% were males, and 43.6% were females; In terms of age, 19.5% were between 18 and 25 years old, 31.7% between 26 and 33, 25.5% between 31 and 35, 14.2% between 36 and 40, 6.2% between 41 and 45, 2.0% between 46 and 50, and 0.8% over 50. About the educational background of the participants, 0.8% had a middle school degree or below, 5.1% with high school degree, 22.1% with junior college degree, 53.0% with an undergraduate degree, and 19.0% with a master degree or above. In terms of their working status, 67% of participants were ordinary staff, 18.6% first-line managers, 12.0% middle managers and 2.4% senior managers. For the length of service, 8.8% were 1 year or less, 27.8% between 1 and 3 years, 28.3% between 3 and 5 years, 21.2% between 5 and 10 years, 13.9% over 10 years.

### Measurement

#### Psychological Capital

We adopted the PsyCap questionnaire (PCQ) of Luthans et al. (2007a) to assess psychological capital. This scale includes four dimensions: efficacy, hope, optimism, and resilience, with 24 items in total. A sample items is “I feel confident analyzing a long-term problem to find a solution.” The Cronbach’s alpha coefficient of four dimensions – efficacy, hope, optimism, and resilience was 0.88, 0.79, 0.73, and 0.72.

#### Leader-Member Exchange

We adopted the leader-member exchange scale of Graen and Uhl-Bien (1995) to measure the quality of exchange relations between leaders and their followers. This scale has totally 7 items. A sample item is “My leader understands my job problems and needs.” Cronbach’s alpha for this scale was 0.83.

Some researchers believed that leader-member exchange is a multi-dimensional construct including affection, contribution, loyalty, and professional regard (Duncan and Herrera, 2014). Other researchers believed that leader-member exchange is a single dimensional continuum from low-quality to high-quality which can reflect the stand or fall of the working relationship between leaders and their followers (Graen and Uhl-Bien, 1995). The low-quality exchange is the social exchange solely based on the employment contract which is called out-group exchange. The high-quality exchange is the material and non-material social exchange beyond formal job description which is called in-group exchange. The current study focused on the quality of leader-member exchange relationship, rather than the content of exchange. So the leader-member exchange scale of Graen and Uhl-Bien (1995) was adopted for measurement.

#### Leader’s Positive Emotions Perceived by Followers

We adopted the positive affect scale in the positive and negative affect scale (PANAS) of Watson et al. (1988) to assess leaders’ positive emotions perceived by followers. The scale includes 10 positive words (such as “exciting,” “passionate” and so on). Followers were asked to evaluate their leaders’ positive emotional states they perceived. Cronbach’s alpha for this scale was 0.83.

Researchers have compiled some scales to measure individual differences of emotional contagions, such as the Questionnaire Measure of Emotional Empathy, the Interpersonal Reactivity Index, and so on. However, these scales focus on measuring individuals’ sensitivity to emotional contagion, rather than the effect of emotional contagion. The effect of emotional contagion is subject to leaders’ emotional expression and followers’ sensitivity, which is a result of interaction between leaders and followers. In order to measure the effect of emotional contagion better, this study revised the PANAS of Watson et al. (1988): First, PANAS is a self-report scale for measuring one’s emotional states. Because this research was designed to measure whether followers are affected by their leaders’ emotions, that is, the effect of emotional contagion, the instruction was modified to ask followers to evaluate their leaders’ emotional state they perceived. Second, because psychological capital belongs to positive psychological resources, we took the negative emotions in the questionnaire away and only measured the positive emotions.

### Analytical Approach

We adopted different self-rating questionnaires for leaders and their followers, respectively. Questionnaires for the leaders include The PCQ and questionnaires for the followers include PCQ, Leader-Member Exchange Scale, and positive affect scale.

Leaders’ psychological capital was a level-2 variable at the group level. Followers’ psychological capital, leader-member exchange and leader’s positive emotions perceived by followers were level-1 variables at the individual level. The mediation hypotheses we proposed need to be tested with a 2-level nested data. There was a cross-level mediation-lower mediator model, namely the 2-1-1 model. The nature of data and the result of the intra-class correlation coefficient (ICC) will indicate the applicability of using multilevel model. In this study, *ICC*⁢(1)⁢=⁢τ_00_⁢/⁢τ_00_+σ^2^ = 0.11/ (0.11+0.12) = 0.48>0.06, it means that factors in group level explained 47.8% variation of dependent variable. Therefore, it is necessary to establish a multilevel model. In order to get higher statistical power and more accurate estimates of mediating effects, we adopt both multilevel model (MLM) and multilevel structural equation model (MSEM) to test the hypotheses, as Fang et al. (2014) suggested.

SPSS20.0 was used to conduct description statistics, correlation analysis, exploratory factor analysis, and reliability analysis. Mplus 7.0 was used to conduct confirmatory factor analysis and MSEM analysis. By using software HLM 6.08, we established the multilevel mediation effect model. In the modeling process, we centered level 1 mediators according to group means and put the group means in the level 2 intercept equation to separate between-group mediation effect and within-group mediation effect so as to estimate multilevel mediation effect accurately (Fang et al., 2010). Although the educational background has a positive correlation with follower’s psychological capital, there is no evidence that the inclusion of such control variable is able to provide more accurate estimates of the relationship between leaders’ and their followers’ psychological capital. Considering the proposal of Becker et al. (2016), we avoided including demographic variables in the model. The missing data were filled through the EM algorithm.

### Assessment of Common Method Variance

Harman’s One-Factor Test was adopted to test the common method variance of followers’ psychological capital, leader-member exchange, and positive affect scale (Malhotra et al., 2006). The goodness of fit index of one-factor model is as following: *χ*^2^ = 1416.87, *df* = 135, NNFI = 0.52, CFI = 0.57, SRMR = 0.12. The goodness of fit index of three-factor model is as following: *χ*^2^ = 691.58, *df* = 132, NNFI = 0.79, CFI = 0.82, SRMR = 0.08. The goodness of fit index of six-factor model (divided psychological capital into 4 dimension) is as following: *χ*^2^ = 409.03, *df* = 120, NNFI = 0.88, CFI = 0.91, SRMR = 0.07. The goodness of fit indexes of both the three-factor model and the six-factor model were far better than that of the one-factor model, while that of the six-factor model was better than that of the three-factor model. The result suggested that common method variance had little influence on this research.

## Results

### Descriptive Statistics of Variables

Descriptive statistics (see Table 1) showed: Followers’ psychological capital was positively related to followers’ leader-member exchange and perceived positive emotions.

**TABLE 1:** Description statistics.

**variables**	***M***	***SD***	**1**	**2**	**3**	**4**	**5**	**6**	**7**	**8**
(1) psychological capital of leaders^a^	4.20	0.46	1							
(2) sex of followers^b^	0.56	-	–0.130	1						
(3) age of followers^c^	2.49	1.17	0.093	0.059	1					
(4) length of service of followers	2.89	1.12	0.335	0.065	0.786^∗∗^	1				
(5) education background of followers	3.79	0.82	0.493^∗∗^	0.003	0.051	0.057	1			
(6) psychological capital of followers	3.96	0.47	0.717^∗∗^	0.047	–0.082	0.064	0.414^∗∗^	1		
(7) leader-member exchange	3.83	0.56	0.583^∗∗^	0.063	0.089	0.138^*^	0.253^∗∗^	0.489^∗∗^	1	
(8) leader’s positive emotions perceived by followers	3.02	0.64	0.112	0.020	0.009	–0.012	–0.145^∗∗^	0.082	0.263^∗∗^	1

^*a*^Psychological capital of leaders was data in work group level which is without correlation coefficient with variables in individual level, *N* = 32. ^*b*^1 = male, 0 = female, the mean represented the proportion of male. ^*c*^Age used a 7-point scale. Length of service and education background used a 5-point scale, *N* = 321. ^*^p < 0.05; ^∗∗^*p* < 0.01.

### The Test of Multilevel Mediation Effect Models

The result of MLM as follows (see Table 2): First, we test the null model (M_0_) to examine the percentage of variation in the dependent variable that can be interpreted by between-group variations, namely ICC. In this study, *ICC*⁢(1)⁢=⁢τ_00_⁢/⁢τ_00_+σ^2^ = 0.11/ (0.11+0.12) = 0.48, it means that factors in group level explained 48% variation of dependent variable. Random effects of between-group variance were significant (*τ*_00_ = 0.11, χ^2^(31) = 308.08, *p* < 0.001), which suggested that there was significant variation has not been explained and we need to add predictor variables to explain them.

**TABLE 2:** The test of multilevel multiple mediation effect.

**Models**	**Parameter estimation**						
	
	***γ*_00_**	***γ*_01_**	***γ*_02_**	***γ*_03_**	***γ*_10_**	***σ*^2^**	***τ*_00_**
M_0_: null model	3.96^∗∗∗^					0.12	0.11^∗∗∗^
L1:Fpsycap*_*ij*_* = *β*_0_*_*j*_* + *r*_*ij*_							
L2:*β*_0j_ = *γ*_00_ + *μ*_0_*_*j*_*							

M_1_: psychological capital of leaders → psychological capital of followers	1.69^∗∗^	0.54^∗∗∗^				0.12	0.05^∗∗∗^
L1:Fpsycap*_*ij*_* = *β*_0_*_*j*_* + *r*_*ij*_							
L2:_0j_ = *γ*_00_ + *γ*_01_^*c*^ (Lpsycap) + *μ*_0_*_*j*_*							

M_2_: psychological capital of leaders → leader-member exchange	1.84^∗∗^	0.48^∗∗∗^				0.21	0.06^∗∗∗^
L1: LMX*_*ij*_* = *β*_0_*_*j*_* + *r*_*ij*_							
L2: *β*_0j_ = *γ*_00_ + *γ*_01_^*a*⁢1^ (Lpsycap) + *μ*_0_*_*j*_*							

M_3_: psychological capital of leaders → leaders’ positive emotions perceived by followers	2.55^∗∗^	0.12				0.24	0.18^∗∗∗^
L1: PA*_*ij*_* = *β*_0_*_*j*_* + *r*_*ij*_							
L2: *β*_0j_ = *γ*_00_ + *γ*_01_^*a*⁢2^ (Lpsycap) + *μ*_0_*_*j*_*							

M_4_: psychological capital of leaders, leader-member exchange, leaders’ positive emotions perceived by followers → psychological capital of followers	0.91	0.32^∗∗^	0.47^∗∗^	−0.03		0.11	0.04^∗∗∗^
L1: Fpsycap*_*ij*_* = *β*_0_*_*j*_* + *β*_1_*_*j*_* (LMX)+*β*_2_*_*j*_* (PA) + *r*_*ij*_							
L2: *β*_0j_ = *γ*_00_ + *γ*_01_^c′^ (Lpsycap) + *γ*_02_ (MLMX) + *γ*_03_ (MPA) + *μ*_0_*_*j*_*							
*β*_1j_ = *γ*_10_^*b*⁢1^					0.24^∗∗^		
*β*_2j_ = *γ*_10_^*b*⁢2^					−0.03		

*(1) *σ*^2^ means residual error of layer 1 and *τ*_00_ means residual error of intercept, namely *μ*_0j_; (2) LMX means leader-member exchange. PA means leaders’ positive emotions perceived by followers. Lpsycap means psychological capital of leaders. Fpsycap means psychological capital of followers. MLMX means leader-member exchange aggregated into group level. MPA means leaders’ positive emotions perceived by followers aggregated into group level. (3) Variables in layer 1 were centered according to group means. ^∗∗^*p* < 0.01; ^∗∗∗^*p* < 0.001.*

Next, we examined the direct effect *c* of independent variable *X*_j_ on dependent variable *Y*_ij_ (M_1_). The results showed that leaders’ psychological capital had significant effects on followers’ psychological capital (*γ*_01_^*c*^ = 0.54, *t*(30) = 5.79, *p* < 0.001). The between-group variance of followers’ psychological capital dropped from 0.11 (M_0_) to 0.05 (M_1_). This decreasing random effect verified Hypothesis 1.

Then, we examined the direct effect *a* of independent variable *X*_j_ on mediator *M*_*ij*_ (M_2_, M_3_). The results showed that leaders’ psychological capital had significant effects on leader-member exchange (*γ*_01_^a^ = 0.48, *t*(30) = 4.15, *p* < 0.001). However, the direct effect of leaders’ psychological capital on leaders’ positive emotions perceived by followers was not significant (*γ*_01_^a^ = 0.12, *t*(30) = 0.65, *p* > 0.05).

At last, we examined the effect *c’* of the independent variable and the effect *b* of mediator on the dependent variable (M_4_). The results showed that leader-member exchange played a positive role in promoting the followers’ psychological capital (*γ*_10_^b^ = 0.24, *t*(315) = 5.85, *p* < 0.01). The between-group effect of leader-member exchange had a significant effect on followers’ psychological capital (*γ*_02_ = 0.47, *t*(28) = 3.13, *p* < 0.01). However, leaders’ positive emotions perceived by followers had no significant influence on the followers’ psychological capital (*γ*_20_^b^ = −0.03, *t*(315) = −0.66, *p* > 0.05). The between-group effect of leaders’ positive emotions perceived by followers on the followers’ psychological capital was not significant (*γ*_03_ = −0.03, *t*(28) = −0.35, *p* > 0.05). The influence of leaders’ psychological capital on their followers’ psychological capital was decreased but still significant (*γ*_01_^c′^ = 0.32 < *γ*_01_^c^ = 0.54, *t*(28) = 2.97, *p* < 0.01).

Given these results, it is reasonable to conclude that leader-member exchange is the mediator between psychological capital of leaders and followers, but leaders’ positive emotions perceived by followers have no significant mediating effect in the relationship of leaders’ and their followers’ psychological capital.

In order to get more accurate estimates of mediating effects, the result of MSEM as follows (see Table 3): The mediation effect of leaders’ psychological capital on their followers’ psychological capital through leader-member exchange was significant [*a* × *b* = 0.28, 90% CI (0.10, 0.46)]. The mediation effect of leaders’ psychological capital on their followers’ psychological capital through emotional contagion was insignificant [*a* × *b* = −0.01, 90% CI (−0.04, 0.03)].

**TABLE 3:** Multilevel structural equation model result for path *a*, path *b*, and the indirect effect.

**Mediation**	***a* path**	***b* path**	**Mediation effect (90% CI)**
Fpsycap→LMX→Fpsycap	0.48^∗∗∗^ (0.11)	0.59^∗∗^ (0.18)	0.28 (0.10, 0.46)
Fpsycap→PA→Fpsycap	0.12 (0.16)	−0.04 (0.12)	−0.01 (−0.04, 0.03)

*(1) LMX means leader-member exchange. PA means leaders’ positive emotions perceived by followers. Lpsycap means psychological capital of leaders. Fpsycap means psychological capital of followers. CI means confidence interval. (2) Standard errors are shown in parentheses. ^∗∗^*p* < 0.01; ^∗∗∗^*p* < 0.001.*

## Discussion

### Mechanism of Leaders’ Psychological Capital Effects on Followers’ Psychological Capital

Based on the studies of Walumbwa et al. (2010) and Ren et al. (2013), this study investigated how leaders’ psychological capital influences their followers’ psychological capital by proposing and testing a multilevel multiple mediation model. The results suggested that the effect of leaders’ psychological capital on their followers is a product of social exchange, rather than emotional contagion.

Social exchange theory emphasizes that social interaction between people is a process of mutual exchange by which people establish and maintain most of the economic and social relations with others. In social exchange, they follow the norm of reciprocity and then form the sense of obligation, reciprocity, and trust in relationships accordingly (Walumbwa et al., 2011; Cook et al., 2013). In organizations or work teams, economic exchange such as wages and salaries happens everywhere. At the same time, psychological exchange such as support and trust also happens.

Social exchange of psychological characteristics between team leaders and their followers, for instance, the exchange of psychological capital, is a prerequisite for leaders to exert their functions normally. It is also an inevitable result of team operation since a team cannot develop without effective exertion of leaders’ function. At different stages of development, team leaders take different functions. No matter how leader’s role changes, it involves two aspects: improving followers’ knowledge or skills and promoting followers’ positive psychological qualities for a harmonious organization atmosphere (Kozlowski and Bell, 2003; Mathieu et al., 2014). Therefore, leaders need to keep a high-quality exchange relationship with their followers and to influence their followers by their own positive psychological traits.

From the team members’ point of view, those who can establish a high-quality exchange relationship with leaders will get more support, work flexibility and trust (Graen and Uhl-Bien, 1995). According to the norm of reciprocity in social exchange, these in-group followers will have pressure to improve their work performance for return. In the social exchange between leaders and members in organizations, leaders often occupy the predominant position, so that they can make requests to their followers as the return, further allocate organizational resources and rights. In this circumstance, it is necessary for followers to repay their leaders with the purpose of maintaining a high-quality exchange relationship and gaining more resources. Therefore they will be more motivated at work and tend to show a positive state with efficacy, optimism, resilience, and hope.

The leader was generally considered a role model for team followers. So both leaders’ words and deeds will influence followers’ thinking and acting. If a leader’s psychological capital is abundant, team members he or she led are more likely to observe and to experience the positive consequences brought by leaders’ psychological capital. This progress helps to stimulate followers to form optimistic expectations and stronger motivation at work (Yammarino et al., 2008). Consequently, followers will develop positive psychological qualities such as efficacy, optimism, resilience, and hope, too.

Chinese culture attaches great importance to “gratitude” and “return,” especially stressing reciprocity in interaction. In ancient times, there were sayings such as “Gentleman must reciprocate for favors; A drop of water in need shall be returned with a spring in deed.” and “One should repay with gratitude for help and encouragement by a superior.” These sayings reflect the norm of reciprocity in social exchange and emphasize mutual trust and mutual benefit as the basis of social relations and social ethics. Under the background of Chinese society and culture, the current research showed that when team members perceive the attention, trust, care, and understanding of their leader, they tend to feel obligated to return the leader’s appreciation, so as to show more activity and psychological capital.

In explaining how leaders’ psychological capital influences their followers’ psychological capital, once it was thought that leaders’ psychological capital influence their followers through emotional contagion. That is, a leader with high levels of psychological capital can affect their followers by positive emotional expressions in that it makes the followers experience more positive psychological qualities and positive emotions (Bono and Ilies, 2006; Walumbwa et al., 2010; Ren et al., 2013). This study did not support this hypothesis, and it may be related to the dynamic characteristics of emotional contagion, or the cultural background of participants.

Emotional contagion is a dynamic process. In interpersonal interaction, deliverers express their emotions through symbols such as facial expressions, movements, voices. Then, their emotions were perceived by observers who will express these emotions again consciously or unconsciously, and feedback to the deliverer, forming a cycle effect. Thus it can be seen that, if the leader’s psychological capital influences followers’ through emotional contagion, the following conditions must be satisfied: Firstly, leader’s psychological capital has been expressed by translating into positive emotional symbols. Secondly, emotional symbols leaders delivered can be perceived by followers. Thirdly, followers internalize leaders’ emotions they perceived and express these emotions or simply imitate them. If any of above conditions is not satisfied, the dynamic chain of emotional contagion will be broken. Complete emotional communication or interaction between leaders and followers will be deterred.

Traditional Chinese organizations are usually set up on the basis of obedience to authority. In that case, followers usually express considerable respect and obedience to their leaders, while leaders are working hard to maintain credibility, permanency, and versatility of their authority. Leaders are often unwilling to express their emotions too much in front of their followers in order to keep their authority figure. In fact, leaders already become a symbol of power because they control over the allocation of resources, the personnel’s future, and the evaluation of followers. Hierarchy between leaders and followers is rigid. Followers respect, obey, and even fear the authority of leaders, while leaders are also very hard to “mingle with” followers. In a task-oriented team environment, followers pay more attention to leaders’ orders or directives rather than leaders’ emotional changes. Therefore, under the background of Chinese culture, emotional contagion theory may be not applicable to explain leaders’ influence on followers.

### Implications

The methods and findings of the current study have certain significances for research on psychological capital.

From a methodological perspective, this study explores the relationship between variables from different levels in organizational context by hierarchical linear models, improving the ecological validity and the explanatory power of conclusion.

From a theoretical perspective, by comparing different assumptions from social exchange theory and emotional contagion theory, this study investigated the influential mechanism of leader’s psychological capital on their followers’ psychological capital, and the results support the social exchange theory. This result not only helps to build up our understanding of the influential mechanism of leader’s psychological capital on their followers but helps us to further understand the positive role of social exchange on individual mentality and organization performance. The finding of this study expanded leadership researches which usually focused on specific leadership style, such as transformational leadership. Unlike that, we paid attention to leaders’ psychological capital and found its positive effect on followers’ psychological capital through leader-member exchange. The results also extended the social exchange theory. Our finding supported Story et al. (2013) conclusion that the quality of the relationship played a more important role in the process of psychological capital’s interaction.

Our findings also give advice on organizational practice. For organizations, it’ necessary to attach great importance to the improvement of leaders’ and followers’ psychological capital by training (Luthans et al., 2010). For leaders, it’s necessary to show high levels of positive psychological capital and develop high-quality relationships with their team members and followers.

### Limitations and Suggestions for Future Research

There are also several limitations can be found in this study which should be noted. First, our research is based on the cross-sectional data, which cannot provide evidence for causation and subject to common method biases. Second, boundary conditions which were important for intervention work were not considered in this study. Third, although the sample size in level 2 reaches the minimum threshold recommended for multilevel analysis (Kreft, 1996), the sample size on level 2 (group level) is not big enough. For future research, we have some suggestions. First, longitudinal studies were recommended for the possible causal inference. Second, besides important mediation variables, moderator variables may be taken into full consideration, such as personality traits. Third, larger sample size is needed to further prove the founding in this study. For another, researchers can design a new study to compare different possible hypotheses (e.g., social learning theory etc.) in explaining the interaction between leaders’ psychological capital and their followers. Last but not least, when researchers review and further explore these relationships, the cultural differences should be highly regarded and deeply discussed.

## Conclusion

Leaders’ psychological capital has a positive influence on their followers’ psychological capital. Leader-member exchange is the multilevel mediator between leaders’ psychological capital and followers. The multilevel mediating effect of leaders’ positive emotions perceived by followers is not significant. Based on social exchange theory, this study provides novel insights into the mechanism through which leaders’ psychological capital is related to their followers’. These findings highlight the importance to improve leader s’ psychological capital and develop high-quality leader-member relationship so as to foster their follower s’ psychological capital.

## Ethics Statement

The present study has been approved by the Research Ethics Committee, South China Normal University. All participants provided their consent before completing the measures. The data were collected and analyzed anonymously.

## Author Contributions

QC contributed to developing the theoretical framework, data analysis, organization, and overall writing of the manuscript. YK, JN, WG, JL, and ML contributed to the design, data analysis, and editing of the manuscript.

## Conflict of Interest Statement

The authors declare that the research was conducted in the absence of any commercial or financial relationships that could be construed as a potential conflict of interest.
